# A meta-analysis of the impact of different ALK variants on targeted therapy efficacy in advanced non-small cell lung cancer

**DOI:** 10.3389/fonc.2026.1756464

**Published:** 2026-03-25

**Authors:** Ziye Gu, Zixuan Chen, Qing Lai, Dang Lin

**Affiliations:** Department of Pulmonary and Critical Care Medicine, Affiliated Suzhou Municipal Hospital of Nanjing Medical University, Suzhou, China

**Keywords:** ALK variant, crizotinib, non-small cell lung cancer, progression-free survival, targeted therapy

## Abstract

**Background:**

Anaplastic lymphoma kinase (ALK) fusion is an important therapeutic targets in non-small cell lung cancer (NSCLC). Different ALK variants may affect the efficacy of targeted therapies. This meta-analysis systematically assesses the impact of different ALK variants on the clinical outcomes of ALK TKI treatment.

**Methods:**

By systematically searching PubMed, Embase, and Web of Science databases, we collected relevant studies published from January 1,1994 to September 30, 2025. The relationship between different ALK variations and treatment efficacy was evaluated by combining hazard ratio (HR) and 95% confidence interval (CI). The quality of studies was evaluated using tools such as the Newcastle-Ottawa Scale (NOS) and the Cochrane risk-of-bias tool.

**Results:**

A total of 30 studies involving 2737 patients with ALK-positive NSCLC were included. Comparison between EML4-ALK variant 1 (V1) and variant 3 (V3) showed that V3 was associated with shorter progression-free survival (PFS) in patients receiving ALK TKI treatment (HR = 1.53, 95%CI:1.17-1.99, p=0.002). Subgroup analysis showed that the adverse effect of V3 was more pronounced in patients treated with crizotinib (HR = 1.40, 95%CI: 1.00-1.96, p=0.049), in the first line treatment setting (HR = 1.83, 95%CI: 1.34-2.50, p<0.001), and in those assessed by NGS (HR = 1.67, 95%CI: 1.34-2.08, p<0.001). A significant association was also observed in the brigatinib-treated population (HR = 2.09, 95%CI: 1.33-3.28, p=0.001), although this finding was based on only two studies. When comparing V3 with non-V3 variants, V3 was associated with significantly worse PFS (HR = 1.78, 95%CI:1.38-2.30, p<0.001). When comparing V1 with non-V1 variants, V1 was associated with significantly better PFS (HR = 0.63, 95%CI:0.44-0.89, p=0.01); however, after excluding V3, no significant difference was found between V1 and other variants. No significant differences were observed between V1 and V3 in overall survival (OS) or objective response rate (ORR).

**Conclusion:**

EML4-ALK v3 may be an important negative prognostic factor for the efficacy of targeted therapy in ALK positive NSCLC. Subgroup analysis indicated that the poor prognosis associated with v3 was particularly evident in patients treated with crizotinib, in the first line setting, and in those assessed by NGS. However, due to limited data on newer generation ALK TKIs and the presence of heterogeneity in some of the comparison groups, definitive conclusions cannot be drawn. Prospective studies with standardized molecular subtyping are still needed before considering clinical stratification based on ALK variant types.

**Systematic Review Registration:**

https://www.crd.york.ac.uk/PROSPERO/view/CRD420251229641, identifier CRD420251229641.

## 1 Introduction

In the 21st century, cancer has become a serious global challenge, profoundly affecting multiple aspects such as society, public health and the economy. Meanwhile, the incidence of cancer worldwide continues to rise. The 2022 data from IARC highlight the continuing severity of cancer as a major public health issue, with nearly 20 million new cases and approximately 9.7 million deaths reported globally (1). Among these, lung cancer is the most common type of cancer and also the main cause of cancer-related deaths (1). According to histopathological criteria, lung cancer falls into two principal categories: non-small cell lung cancer (NSCLC) and small cell lung cancer (SCLC). As the predominant form, NSCLC represents roughly 85% of cases. Its major subtypes include adenocarcinoma, squamous cell carcinoma, and large cell carcinoma (2).

In 1994, ALK was initially identified as a fusion partner of nucleophosphoprotein (NPM-ALK) resulting from a chromosomal translocation in anaplastic large cell lymphoma (3). Subsequently, ALK rearrangements were discovered in many different cancers, including non-small cell lung cancer (NSCLC), colorectal cancer, breast cancer, diffuse large B-cell lymphoma (DLBCL), and others. With the development of molecular detection technology, researchers have found that there are multiple mutant types of ALK variants in NSCLC. The echinoderm microtubule-associated protein-like 4 (EML4) gene is the main ALK fusion partner in NSCLC and was first reported in 2007 (4, 5). The EML4-ALK fusion gene accounts for 3%–6% of lung adenocarcinoma nd is particularly abundant in younger adenocarcinoma patients who have never smoked or are light smokers (6). When the intracellular tyrosine kinase domain of ALK is fused with various EML4 truncations, multiple EML4-ALK variants are generated. The most common of these are EML4 variant 1 (V1) and 3a/b (V3a/b). V1 is characterized by the fusion of EML4 exon 13 with ALK exon 20, while V3a/b is formed by the combination of EML4 exon 6a/b and ALK exon 20 (7, 8). According to the different breakpoint locations of the EML4 gene, EML4-ALK fusion variants can be divided into two categories: short fusion variants and long fusion variants. The former is represented by V3, whose breakpoints are located in EML4 exon 6 or upstream, resulting in truncation or complete deletion of the tandem atypical propeller (TAPE) domain β. The latter covers variants such as V1 and V2, and the breakpoints are usually located downstream of exon 6, thus retaining a relatively intact TAPE domain (9). The structural differences between these two types of variants further endow them with different biological characteristics: compared with the long fusion variant, the short fusion variant has higher protein stability and longer half-life, and is relatively less sensitive to ALK-TKI in vitro (10). In recent years, several retrospective studies have tried to clarify the relationship between different ALK fusion variants and the efficacy of ALK TKIs, but the results have been inconsistent. Most studies confirm that compared with other variants, v3 is associated with shorter progression-free survival (PFS), suggesting a poorer prognosis (11, 12). However, multiple studies revealed no statistically significant differences in ALK TKI treatment outcomes between variants (13–15).

Therefore, this meta-analysis explored the influence of distinct ALK variants on ALK TKI treatment outcomes, seeking to provide clinical practice for ALK-positive NSCLC.

## 2 Methods

### 2.1 Study protocol and registration

This review and meta-analysis as conducted in accordance with the PRISMA guidelines. Additionally, the protocol received registration on the PROSPERO platform (CRD420251229641), available at: https://www.crd.york.ac.uk/PROSPERO/view/CRD420251229641.

### 2.2 Data sources and search strategy

The databases of PubMed, Embase, and Web of Science were systematically queried for records from January 1,1994 until September 30, 2025, without any language or study type limitations. The search used a combination of subject headings and free-text terms. Key search terms included: “anaplastic lymphoma kinase”, “ALK”, “variant”, “non-small cell lung cancer”, “ALK inhibitor”, “crizotinib”, “alectinib”, “lorlatinib”, “brigatinib”,”ceritinib”,and related synonyms.

The PICOS framework was used to define the research question and guide the literature search strategy to ensure a comprehensive and unbiased retrieval: the population (P) was restricted to patients with advanced, ALK-positive non-small cell lung cancer; the intervention/exposure (I) referred to specific ALK variant types (e.g., EML4-ALK V1, V3); the comparator (C) was set to compare between different variant types; the primary outcome (O) was pre-specified as progression-free survival (PFS), with secondary outcomes including overall survival (OS) and objective response rate (ORR); eligible study designs (S) consisted of the following: observational studies, single-arm trials, randomized controlled trials, and diagnostic accuracy studies.

### 2.3 Inclusion and exclusion criteria

The inclusion criterias were as follows:

1. Study Types: RCTs, observational studies, single-arm trials, and diagnostic accuracy studies.
2. Study Population: Adult patients (age ≥18 years) with histologically confirmed advanced ALK-positive NSCLC and with specific ALK variant types confirmed by RT-PCR, next-generation sequencing (NGS), or FISH were included.
3. Intervention/Exposure: Patients were treated with ALK TKIs. The included studies had to categorize patients according to the specific ALK variants identified.
4. Outcome indicators: Studies reporting the relationship between different ALK variants and treatment efficacy (e.g., PFS, OS, ORR).

The exclusion criterias were as follows:

1. Case reports, case series (with sample size <10), reviews, systematic reviews, and studies involving *in vitro* experiments.
2. Studies that did not provide data on the association between different ALK mutants and treatment outcomes, and for which individual patient data could not be reconstructed from Kaplan Meier curves.
3. Duplicate publications or studies with overlapping patient populations (only the one with the most complete and recent data was retained).
4. Studies for which the full text or key data could not be obtained.

### 2.4 Study selection and data extraction

Two investigators independently performed study selection and data extraction. The difference is adjudicated by the third researcher to reach a consensus. We use EndNote X9 to manage records and assist with filtering.

For each included study, we collected the following information:

1. Basic study information: first author, publication year, study type, and country.
2. Patient characteristics: sample size, ALK testing method, and distribution of ALK mutants (EML4-ALK V1, V2, V3, and others).
3. Treatment information: type of ALK TKI and line of therapy.
4. Outcome measures: For studies reporting HR and 95% CI, the HR and 95% CI for PFS and OS were directly extracted. For studies providing only Kaplan Meier curves, coordinate point data were digitally extracted from the published Kaplan Meier curves using WebPlotDigitizer, and individual patient time to event data were reconstructed using the IPDfromKM algorithm. Based on the reconstructed IPD, the Cox proportional hazards model was used to calculate the HR and 95% CI for the comparison of V3 versus V1 within each study. The Kaplan Meier curves reconstructed from the IPD were overlaid with the original curves to check for consistency. In addition, the median survival times and survival rates at specific time points (e.g., 12 months, 24 months) obtained from the reconstructed IPD were compared with the data reported in the original article. If the discrepancy was 10% or greater, the IPD data from that study were considered unreliable and were not included in the meta-analysis. ORR data were extracted as percentages (number of events/total sample size).

### 2.5 Quality assessment

For the included retrospective cohort studies, two investigators independently performed quality assessment with the Newcastle-Ottawa Scale (NOS). This scale evaluates eight items within three key domains: selection, comparability, and outcome. A 9-point scoring system was used, and studies obtaining a score of 7 or higher were designated as high-quality. For RCTs, we employed the Cochrane RoB 1.0 tool for risk of bias assessment. This scale assesses aspects including random sequence generation, allocation concealment, blinding of participants and personnel, blinding of outcome assessment, completeness of outcome data, and other sources of bias. For single-arm trials, the MINORS tool was utilized for evaluation. Diagnostic accuracy studies were assessed using the QUADAS-2 instrument.

### 2.6 Statistical analysis

This study employed Stata 17.0 software for statistical analysis. For time-to-event outcomes(e.g., PFS, OS), hazard ratios (HRs) and their 95% confidence intervals (CIs) were extracted from the studies for pooling. For dichotomous outcomes(e.g., ORR), pooled relative risks (RRs) and their 95% CIs were calculated. The Q test and I² statistics were used to evaluate the heterogeneity between studies. When the heterogeneity test results met the following criteria:I²<50% and the p-value of the Q test > 0.10, we considered the heterogeneity between studies to be insignificant and pooled analyses using a fixed-effects model. Otherwise, a random-effects model was utilized. We conducted subgroup analyses—stratified by ALK TKI agent, treatment line, and detection method—to explore the source of heterogeneity. If the number of included studies is sufficient (≥10), funnel plot visual symmetry was assessed, combined with a quantitative evaluation using Egger’s test. An Egger’s test p value < 0.05 suggests the presence of potential publication bias.

## 3 Results

### 3.1 Literature screening process and results

**Figure 1:**
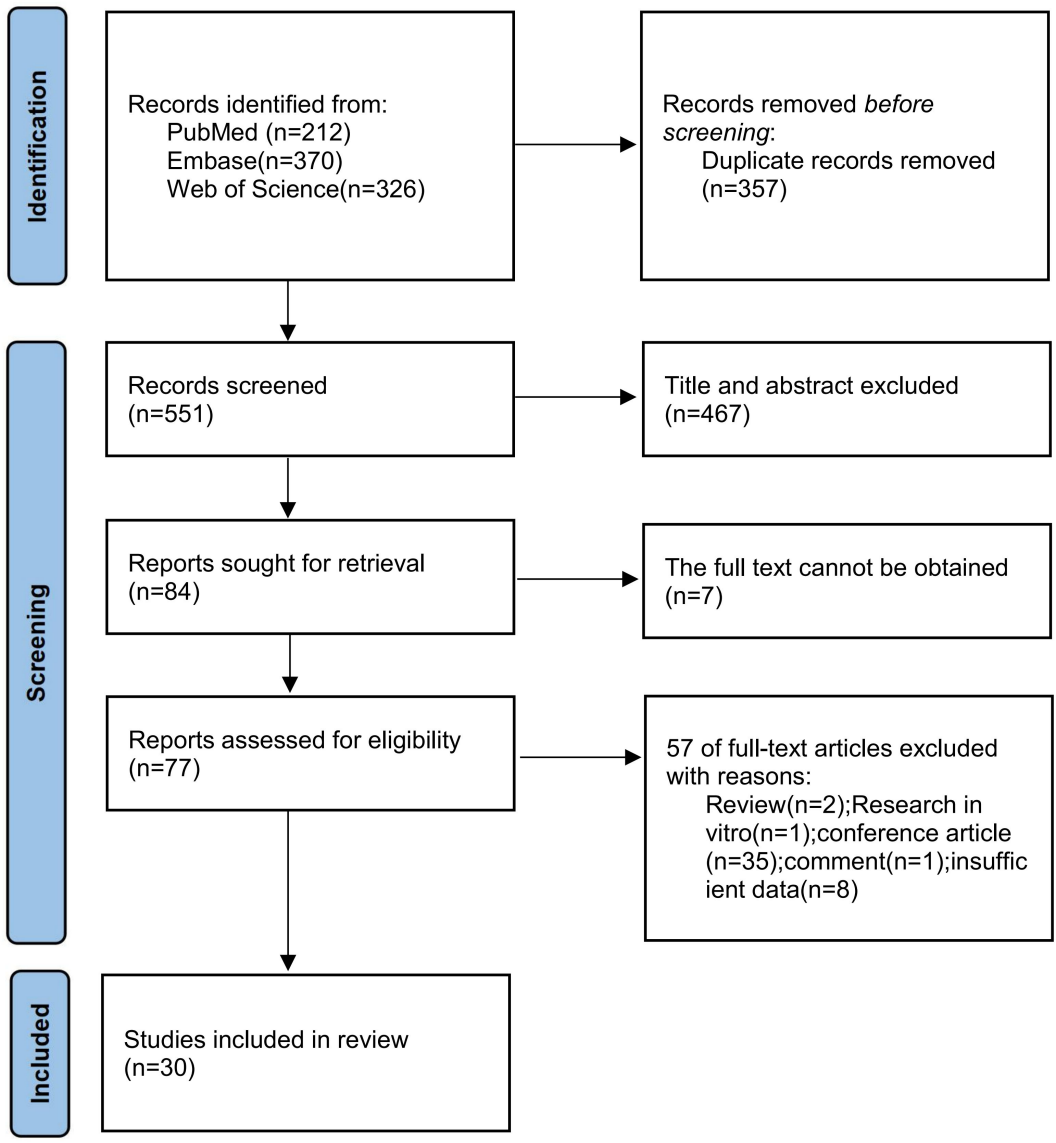
A flowchart of literature screening method.

The initial search identified 908 records. After deduplication and subsequent screening of titles and abstracts, a final set of 30 studies was selected for the analysis. This collection comprised 23 retrospective cohort studies, 3 single-arm trials, 1 diagnostic accuracy investigation, and 3 randomized controlled trials. A flowchart detailing the study selection process is presented in **Figure 1**. The fundamental characteristics of these included studies are summarized in **Tables 1**, **2**.

**Table 1:** Baseline characteristics of included studies and patients.

Study	Country	Time	Study design	n	Variants (V1/V2/V3/others)	Method	Treatment	Treatment line
Zou et al.(2022) (16)	China	2018.09-2022.01	Single-Arm Trial	61	--	RT-PCR,NGS	Alectinib	First-line
Wang et al. (2022) (17)	China	2017.03-2020.09	Retrospective cohort study	101	--	NGS	Crizotinib	First-line
							Alectinib	
Li et al.(2022) (18)	China	2015.01-2020.12	Retrospective cohort study	193	70/25/66/32	NGS	Crizotinib,Alectinib	All lines
Lei et al.(2015) (19)	China	2008.06-2015.03	Retrospective cohort study	61	22/-/18/21	RACE-coupled PCR;sequencing	Crizotinib	All lines
Batra et al.(2021) (20)	India	2013.08-2019.12	Retrospective cohort study	113	40/17/28/6	NGS	ALK TKI	All lines
							Crizotinib	First-line
Li et al.(2021) (21)	China	2012.01-2016.08	Retrospective cohort study	37	--	NGS	Crizotinib	All lines
Tabbò et al.(2022) (22)	Italy	2011-2017	Retrospective cohort study	55	14/-/15/- (Non-v1,v3 26)	NGS	Crizotinib	All lines
							ALK TKI	All lines
Yoshida et al.(2023) (23)	Japan	--	Single-Arm Trial	29	11/-/18/-	NGS	brigatinib	First-line
Camidge et al.(2021) (24)	North America,Europe,Asia	--	RCT	121	55/11/44/11	NGS	brigatinib	First-line
							Crizotinib	
Fu et al.(2024) (25)	China	2014-2020	Retrospective cohort study	59	--	NGS	Second-generation ALK TKI	All lines
Cha et al.(2016) (26)	Korea	2000.03-2015.02	Retrospective cohort study	52	20/3/10/19	PNA-mediated PCR	ALK TKI	All lines
Yoshida et al.(2016) (27)	Japan	2007.01-2014.12	Retrospective cohort study	35	19/5/4/7	RT-PCR	Crizotinib	All lines
Qiao et al.(2020) (28)	China	2013.01-2017.07	Retrospective cohort study	135	61/17/51/6	Sanger sequencing	Crizotinib	First-line
Lin et al.(2019) (29)	China	2010.12.31-2017.12.31	Retrospective cohort study	54	23/6/18/7	RT-PCR	Crizotinib	All lines
Lin et al.(2018) (30)	America	2008.01-2017.01	Retrospective cohort study	106	55/-/51/-	Sequencing;NGS;WES	Crizotinib	All lines
							ALK TKI	
Parikh et al.(2024) (31)	America	2017.07-2023.03.31	Retrospective cohort study	287	117/24/84/62	NGS	ALK TKI	First-line
Zou et al.(2025) (32)	China	2014.07-2022.03	Retrospective cohort study	109	--	NGS	Crizotinib	First-line
							Alectinib	
Nakazawa et al.(2024) (33)	America	2005.08.31-2022.12.31	Retrospective cohort study	160	88/-/72/-	NGS	ALK TKI	First-line
Guan et al.(2023) (34)	China	2015.09-2020.12	Retrospective cohort study	52	18/6/19/9	NGS	ALK TKI	All lines
Lee et al.(2021) (35)	Korea	2017.08-2019.05	Diagnostic accuracy study	24	19/-/5/-	Nanowire-based cfDNA assay	Crizotinib	First-line
Song et al.(2022) (36)	China	2013.11.01-2019.04.30	Retrospective cohort study	51	23/5/19/4	NGS	Crizotinib	First-line
Woo et al.(2017) (37)	Korea	2011.06-2015.08	Retrospective cohort study	54	18/6/24/6	Peptide Nucleic Acid-mediated(qPCR)	Crizotinib,Alectinib,Ceritinib	All lines
Su et al.(2019) (38)	China	2014.12-2017.05	Retrospective cohort study	110	39/-/41/-	NGS	Crizotinib	All lines
Bearz et al(2023) (39)	Global multi-center	--	RCT	126	26/2/23/13	NGS	Crizotinib	First-line
					20/7/18/17		Lorlatinib	
Ma et al.(2022) (40)	China	2017.02-2021.02	Single-Arm Trial	32	--	NGS	TQ-B3139	All lines
Liu et al.(2022) (11)	China	2014.09-2021.01	Retrospective cohort study	93	26/--/35/--	NGS	Crizotinib	First-line
Christopoulos et al.(2019) (12)	Germany	--	Retrospective cohort study	102	41/11/41/-	NGS,RT-PCR	ALK TKI	All lines
Horn et al.(2019) (41)	America	--	Prospective study	39	17/-/17/-	NGS	Ensartinib	All lines
Camidge et al.(2019) (13)	Global multi-center	--	RCT	222	50/22/49/8	NGS	Crizotinib	First-line
Mitiushkina et al.(2018) (14)	Russia	2012-2017	Retrospective cohort study	64	33/3/16/12	RT-PCR	Crizotinib,Alectinib,Ceritinib	All lines

**Table 2:** Baseline characteristics of included studies and patients.

Study	Treatment	Research	Control	PFS(HR,95%CI)	OS(HR,95%CI)	ORR(%)
Zou et al.(2022) (16)	Alectinib	Long fusion variants	short fusion variants	0.17(0.04-0.68)	--	--
Wang et al.(2022) (17)	Crizotinib	V1	Non-v1	0.62(0.31-1.23)	--	--
	Alectinib			0.87(0.14-5.24)		
Li et al.(2022) (18)	Crizotinib,Alectinib	V3	Non-v3	1.941(1.207-3.12)	--	--
Lei et al.(2015) (19)	Crizotinib	V1	V3	0.80(0.41-1.56)	--	V1:72.70% V3:55.6%
		V1	Non-V1 (except V3)	0.84(0.41-1.71)		
Batra et al.(2021) (20)	ALK TKI	V1	V2	0.86(0.42-1.76)	1.01(0.39-2.65)	--
		V1	V3	1.21(0.56-2.64)	1.11(0.40-3.06)	
	Crizotinib	V1	V2	0.92(0.35-2.40)	0.65(0.15-2.91)	
		V1	V3	0.94(0.36-2.52)	0.86(0.19-3.85)	
Li et al.(2021) (21)	Crizotinib	V3	V1	(0.389-7.857)	(0.339-9.232)	--
		V3	Non-v3 (except v1)	(0.781-4.903)	(0.548-5.121)	
Tabbò et al.(2022) (22)	Crizotinib	V1	Non-v1,v3	0.34(0.15-0.75)	--	--
		V3	Non-v1,v3	0.64(0.30-1.36)		
	ALK TKI	V1	Non-v1,v3	--	0.95(0.31-2.93)	--
		V3	Non-v1,v3		2.12(0.70-6.39)	
Yoshida et al.(2023) (23)	brigatinib	V3	V1	1.95(1.14-3.35)	--	--
Camidge et al.(2021) (24)	brigatinib	V3	V1	2.45(1.07-5.60)	--	V1:84% V3:83%
	Crizotinib			3.42(1.56-7.50)		V1:73% V3:67%
Fu et al.(2024) (25)	Second-generation ALK TKI	Long fusion variants	short fusion variants	5.37(1.64-17.64)	--	--
Cha et al.(2016) (26)	ALK TKI	V1	Non-v1	0.262(0.098-0.699)	--	--
		V2	Non-v2	2.364 (0.305–18.344)		
		V3	Non-v3	1.366 (0.443–4.214)		
Yoshida et al.(2016) (27)	Crizotinib	V1	Non-v1	0.350(0.128-0.929)	--	V1:74% Non-v1:63%
Qiao et al.(2020) (28)	Crizotinib	V1	Non-v1	TTF:0.75(0.72-2.52)	--	V1:70% Non-v1:65.7%
Lin et al.(2019) (29)	Crizotinib	V2	V1	0.99(0.18-5.35)	1.15(0.20-6.75)	V1:42.9% V2:66.7% V3:61.1%
Lin et al.(2018) (30)	Crizotinib	V1	V3	1.3(0.85-1.98)	--	--
	ALK TKI	V1	V3	1.45(0.88-2.38)		
Parikh et al.(2024) (31)	ALK TKI	V3	V1	TTD:1.29(0.83-2.01)	2.34(0.98-5.58)	--
Zou et al.(2025) (32)	Crizotinib	Long fusion variants	short fusion variants	0.65(0.37-1.13)	--	--
	Alectinib			0.30(0.12-0.74)		
Nakazawa et al.(2024) (33)	ALK TKI	V3	Non-v3	1.52(1.03-2.25)	--	--
Guan et al.(2023) (34)	ALK TKI	V3	V1	3.396(1.186-9.725)	3.665(0.949-14.149)	--
Lee et al.(2021) (35)	Crizotinib	V3	V1	3.1(0.8-11.6)	13.5(1.4-32.3)	--
Song et al.(2022) (36)	Crizotinib	V3	Non-v3	1.72(0.93-3.18)	1.73(0.82-3.65)	--
Woo et al.(2017) (37)	Crizotinib,alectinib,ceritinib	V3	Non-v3	4.34(1.67-11.27)	--	V3:82.1% Non-v3:70.1%
Su et al.(2019) (38)	Crizotinib	V3,V5	Non-v3,v5	1.63(0.98-2.73)	2.51(1.11-5.68)	--
Bearz et al(2023) (39)	Crizotinib	V3	V1	1.30(0.65-2.57)	--	V1:50.0% V2:50.0% V3:73.9%
	Lorlatinib			2.18(0.73-6.51)		V1:80.0% V2:85.7% V3:72.2%
Ma et al(2022) (40)	TQ-B3139	V3	V1	0.94(0.21-4.2)	--	--
Liu et al.(2022) (11)	Crizotinib	V3	V1	1.85(1.08-3.18)	--	--
Christopoulos et al.(2019) (12)	ALK TKI	V3	V1,V2	2.4(1.03-5.57)	--	--
Horn et al.(2019) (41)	Ensartinib	V3	V1	1.166(0.42-3.26)	--	V1:53% V3:14% Others:58%
Camidge et al.(2019) (13)	Crizotinib	V3	V1	1.124(0.63-2.00)	--	V1:63.6% V2:66.7% V3:45.8%
		V3	V2	0.81(0.39-1.67)		
		V1	V2	0.80(0.39-1.65)		
Mitiushkina et al.(2018) (14)	Crizotinib,Alectinib,Ceritinib	V1	Non-v1	0.95(0.55-1.63)	--	V1:75.8% Non-v1:80.6% Long fusion variants:76.7% short fusion variants:88.2%

### 3.2 Characteristics and quality assessment of included studies

**Figure 2:**
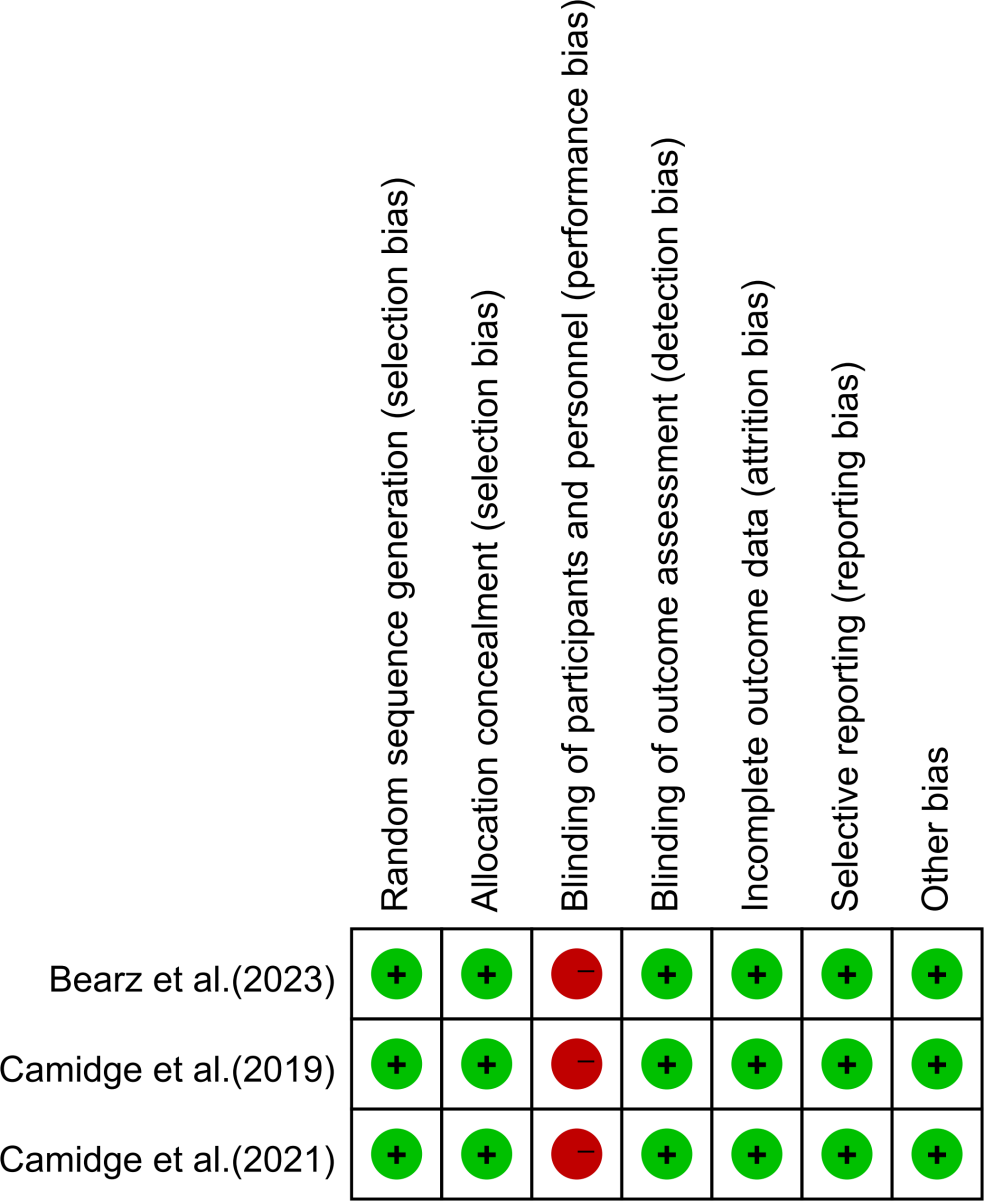
Risk-of-Bias table of RCTs.

The analysis included a cohort of 2737 patients diagnosed with ALK-positive NSCLC. Through detection techniques such as next-generation sequencing and RT-PCR, 786 cases were identified as V3 and 925 cases were identified as V1. Of the 23 included retrospective studies, most showed high methodological quality, with a NOS score of 7 or above, as shown in **Table 3**. 3 study scored 6. The included RCTs were assessed to show a relatively low overall risk of bias (13, 24, 39) (**Figure 2**). The methodological quality of the three included single-arm studies was assessed using the MINORS scale, which consists of 8 items (total score: 16), as shown in **Table 4**. The assessment results showed that the study by Yoshida cored 15, the study by Zou scored 12, and the study by Ma scored 14 (16, 23, 40). Diagnostic accuracy studies were evaluated using the QUADAS-2 tool, and the results showed a low risk of bias in all domains such as patient selection, index testing, reference standards, and flow and timing (35).

**Table 3:** Risk-of-bias table of the retrospective study.

Study	NOS score	Selection (Maximum of 4 stars)	Comparability (Maximum of 2 stars)	Outcome (Maximum of 4 stars)
Wang et al.(2022) (17)	8	4	2	2
Li et al.(2022) (18)	8	4	2	2
Lei et al.(2015) (19)	7	4	1	2
Batra et al.(2021) (20)	7	4	1	2
Li et al.(2021) (21)	9	4	2	3
Tabbò et al.(2022) (22)	8	4	2	2
Fu et al.(2024) (25)	7	4	1	2
Cha et al.(2016) (26)	6	4	0	2
Yoshida et al.(2016) (27)	7	4	1	2
Qiao et al.(2020) (28)	7	4	1	2
Lin et al.(2019) (29)	8	4	2	2
Lin et al.(2018) (30)	7	4	1	2
Parikh et al.(2024) (31)	7	4	1	2
Zou et al.(2025) (32)	7	4	1	2
Nakazawa et al.(2024) (33)	8	4	2	2
Guan et al.(2023) (34)	8	4	2	2
Song et al.(2022) (36)	8	4	2	2
Woo et al.(2017) (37)	8	4	1	3
Su et al.(2019) (38)	7	4	1	2
Liu et al.(2022) (11)	7	4	1	2
Christopoulos et al.(2019) (12)	7	4	1	2
Horn et al.(2019) (41)	6	4	0	2
Mitiushkina et al.(2018) (14)	6	4	0	2

**Table 4:** Risk-of-Bias table of the single-arm trial.

Criteria	Yoshida et al.(2023) (23)	Zou et al.(2022) (16)	Ma et al.(2022) (40)
A clearly stated aim	2	2	2
Inclusion of consecutive patients	2	2	2
Prospective collection of data	2	0	2
Endpoints appropriate to the aim of the study	2	2	2
Unbiased assessment of the study endpoint	2	2	2
Follow-up period appropriate to the aim of the study	2	2	2
Loss to follow up less than 5%	2	2	2
Prospective calculation of the study size	1	0	0

### 3.3 Impact of different ALK variants on the efficacy of ALK TKIs

#### 3.3.1 Primary outcome (PFS)

**Figure 3:**
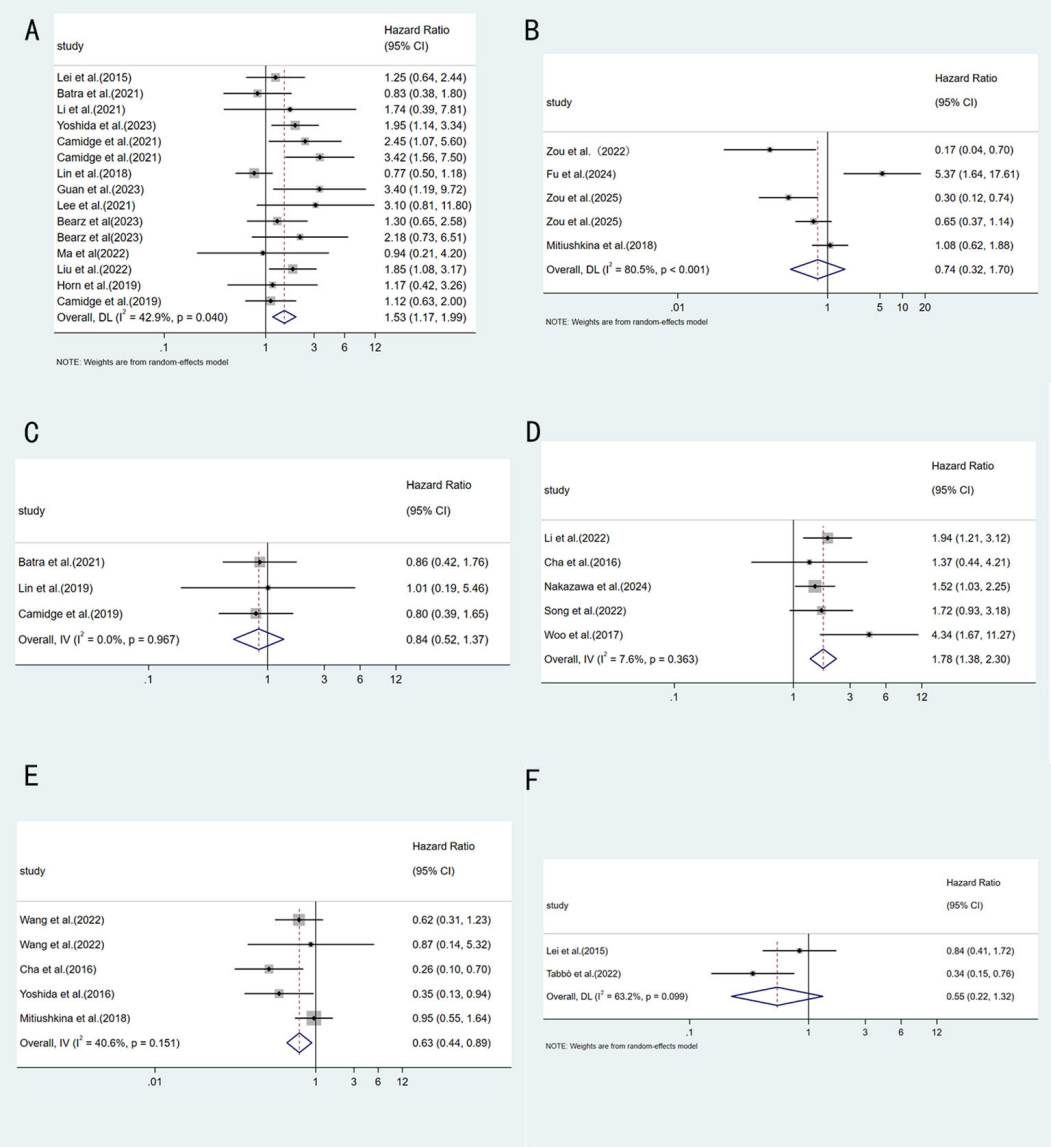
Forest plot comparing PFS between different ALK variants: **(A)** EML4-ALK V3 vs V1; **(B)** Long fusion variant vs short fusion variant; **(C)** EML4-ALK V1 vs V2; **(D)** EML4-ALK V3 vs non-V3; **(E)** EML4-ALK V1 vs non-V1; **(F)** EML4-ALK v1 vs non-V1, V3.

This analysis focused on the association between different ALK fusion variants and PFS. 13 of the included studies directly compared ALK TKI treatment outcomes in patients with V1 and V3. The pooled results showed that V3 patients had a significantly increased risk of disease progression compared with V1 patients (HR = 1.53, 95%CI:1.17-1.99, p=0.002), as shown in **Figure 3A**. This pooled analysis had moderate heterogeneity (I^2^ = 42.9%). In addition, 4 studies evaluated the effects of long fusion variants and short fusion variants on ALK TKIs efficacy, revealing that long fusion variants were associated with prolonged PFS (HR = 0.74, 95%CI:0.32-1.70, p=0.477) (**Figure 3B**). In contrast, no statistically significant difference was observed between V1 and V2 (HR = 0.84, 95%CI:0.52-1.37, p=0.493) (**Figure 3C**). When comparing V3 with non-V3 variants, V3 was associated with significantly worse PFS (HR = 1.78, 95%CI:1.38-2.30, p<0.001) (**Figure 3D**). Conversely, comparison of V1 with non-V1 variants showed that V1 was associated with significantly longer PFS (HR = 0.63, 95%CI:0.44-0.89, p=0.01) (**Figure 3E**). After excluding V3, comparison between V1 and other variants showed no significant difference (pooled HR = 0.55, 95%CI: 0.22-1.23, p=0.179) (**Figure 3F**).

#### 3.3.2 Secondary outcomes (OS, ORR)

**Figure 4:**
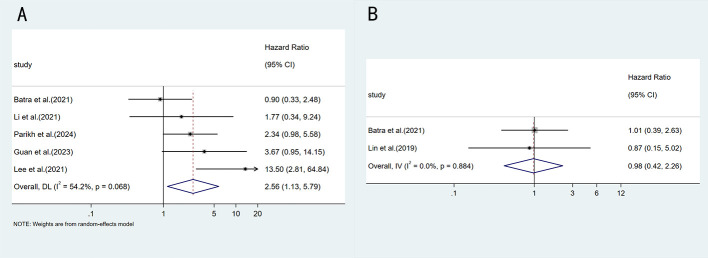
Forest plot comparing OS between different ALK variants: **(A)** EML4-ALK V3 vs V1; **(B)** EML4-ALK V1 vs V2.

In terms of overall survival, there is limited data available for analysis. This study included 6 reports that analyzed the effects of V3 and V1 on the efficacy of ALK TKIs. Analysis using a random-effects model showed that the V3 variant was associated with worse OS, but this did not reach statistical significance (HR = 1.99, 95%CI: 0.94-4.22, p=0.071) (**Figure 4A**). Similarly, no significant difference in OS was found between V1 and V2 (HR = 0.98, 95%CI: 0.42-2.26, p=0.955) (**Figure 4B**).

**Figure 5:**
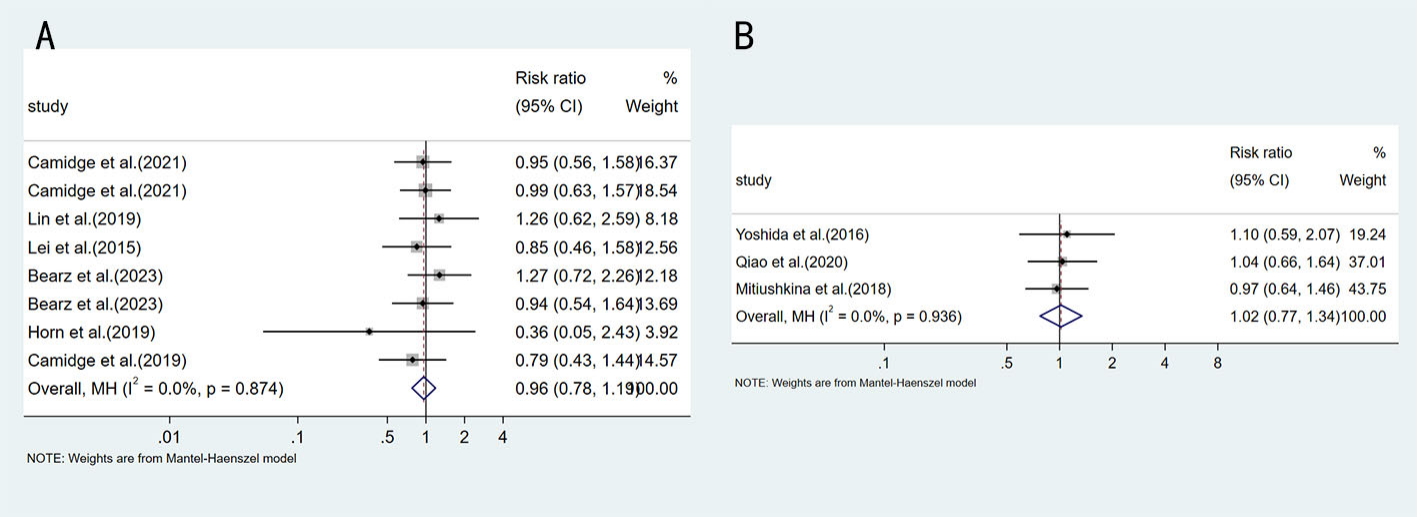
Forest plot comparing ORR between different ALK variants: **(A)** EML4-ALK V1 vs V3; **(B)** EML4-ALK V1 vs non-V1.

Regarding tumor response, when comparing EML4-ALK V3 and V1 (RR = 0.96, 95%CI:0.78-1.19, p=0.716), or when comparing V1 and non-V1(RR = 1.02, 95%CI:0.77-1.34, p=0.894) (**Figure 5**), no statistically significant differences in objective response rate (ORR) were observed.

#### 3.3.3 Subgroup analysis

Given the significant difference and the substantial heterogeneity (I^2^ = 42.9%) between V3 and V1 in PFS, we performed subgroup analyses to explore potential contributing factors.

**Figure 6:**
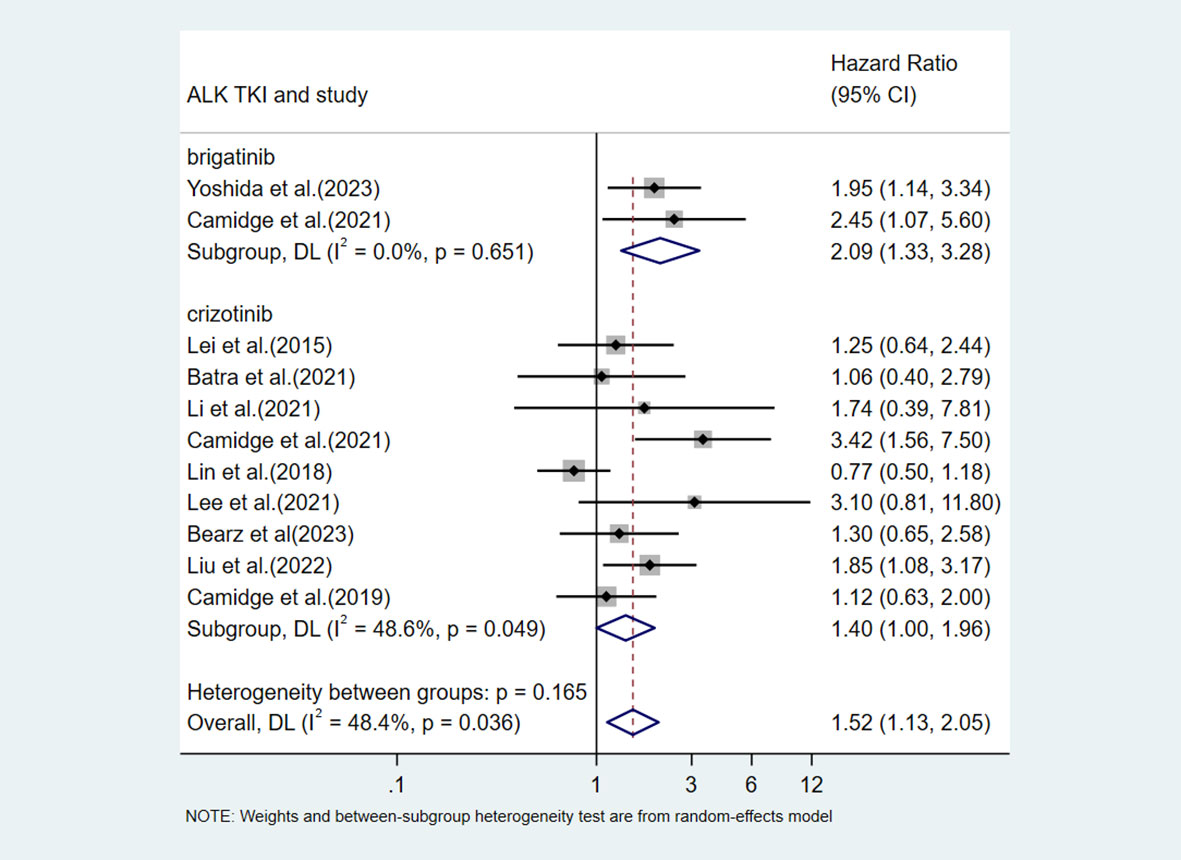
Subgroup analysis of the impact of V3 vs V1 on PFS stratified by ALK TKI type (crizotinib vs brigatinib).

First, we conducted a subgroup analysis based on the type of ALK TKI used. Among the 30 included studies, three studies provided data on alectinib treatment, but the data were reported in a format that could not be directly incorporated into this subgroup analysis comparing V3 and V1. Among the studies with available data for direct comparison between v3 and v1, only one reported data on lorlatinib; therefore, it was not included in the subgroup analysis. The results showed that in the crizotinib group, v3 was associated with worse PFS compared with v1 (HR = 1.40, 95%CI: 1.00-1.96, p=0.049) (**Figure 6**), and moderate heterogeneity was observed within this subgroup (I^2^ = 48.6%). In the brigatinib-treated subgroup, V3 was associated with shorter PFS (HR = 2.09, 95%CI: 1.33-3.28, p=0.001). But given that this finding was based on only two studies, it should be considered exploratory.

**Figure 7:**
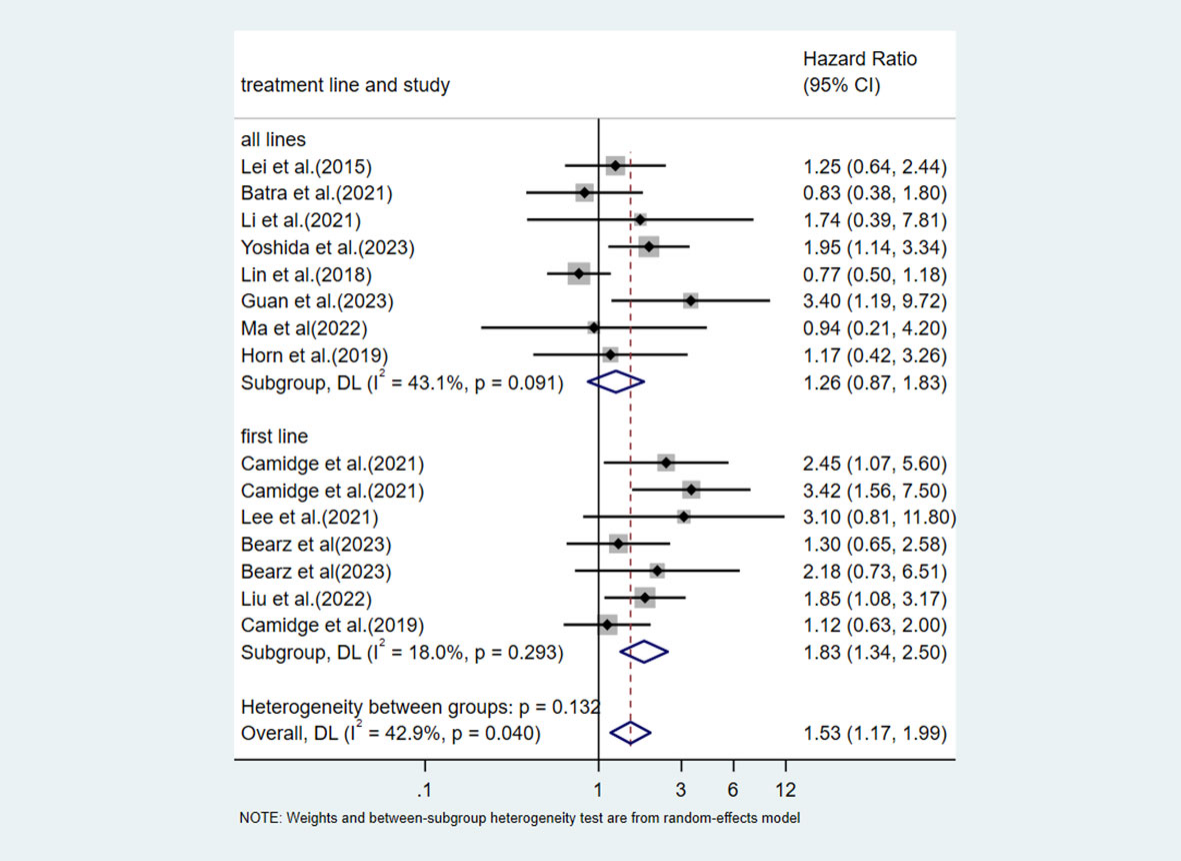
Subgroup analysis of the impact of V3 vs V1 on PFS stratified by ALK TKI treatment lines (first-line treatment vs all lines treatment).

Next, we performed a subgroup analysis according to the line of ALK TKI treatment. In patients receiving treatment across all lines (mixed first-line and later-line therapy), V3 showed a trend toward worse PFS compared with V1, but this did not reach statistical significance (HR = 1.26, 95%CI:0.87-1.83, p=0.277) (**Figure 7**). In patients receiving first-line treatment (HR = 1.83, 95%CI:1.34-2.50, p<0.001) (**Figure 7**), V3 demonstrated a statistically significant PFS disadvantage.

**Figure 8:**
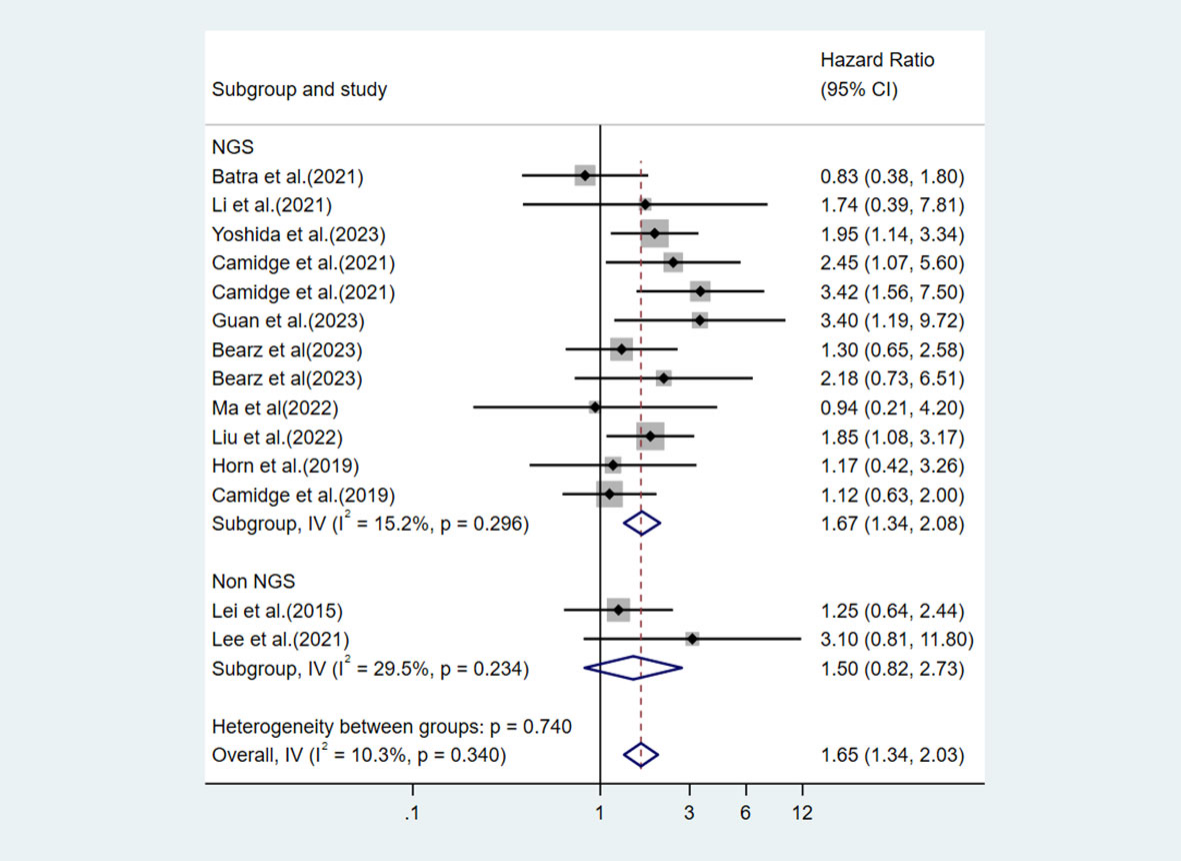
Subgroup analysis of the impact of V3 vs V1 on PFS stratified by ALK mutation detection methods (NGS vs non-NGS).

Finally, we conducted a subgroup analysis based on the mutation detection method used. One study used both NGS and non-NGS methods and did not provide stratified data, so the study was excluded. In the five studies using NGS, V3 was associated with significantly worse PFS (HR = 1.67, 95%CI:1.34-2.08, p<0.001)(**Figure 8**). In the two studies using non-NGS methods, the pooled effect size showed that the difference in PFS between V3 and V1 was not statistically significant (HR = 1.50, 95%CI:0.82-2.73, p=0.185) (**Figure 8**).

#### 3.3.4 Sensitivity analysis and publication bias

**Figure 9:**
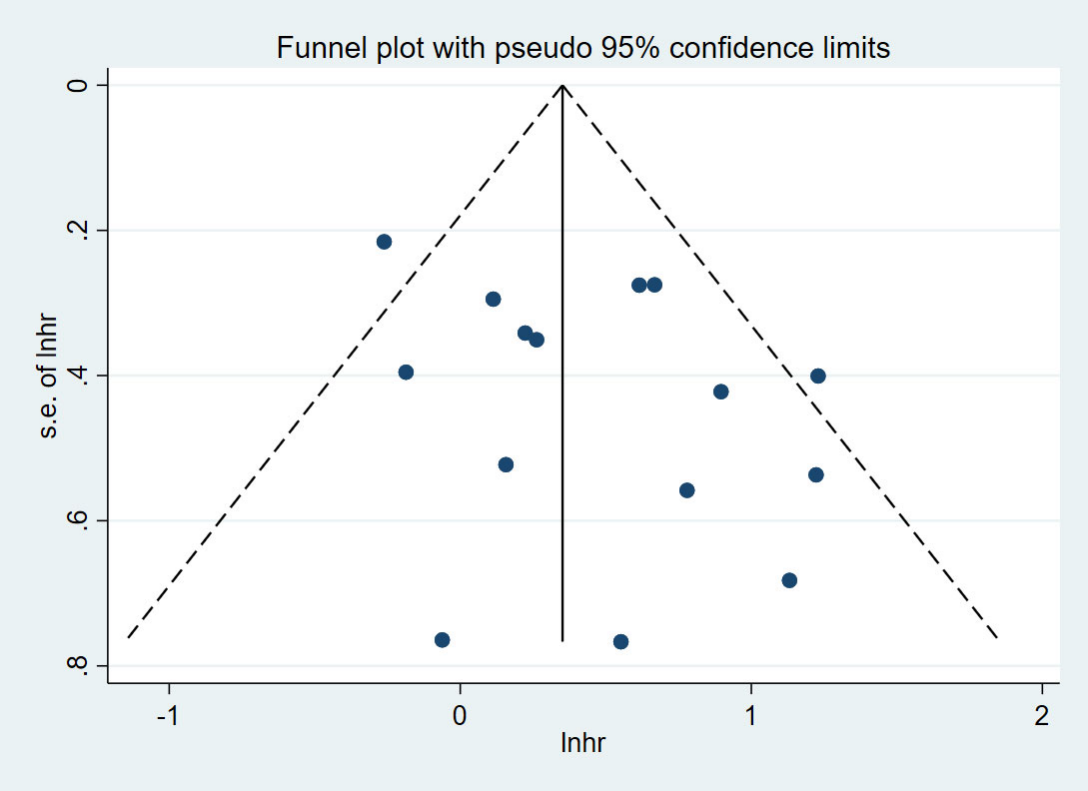
Funnel plot for assessment of publication bias in the PFS between V3 and V1.

To assess the robustness of the primary findings, a sensitivity analysis was conducted by removing individual studies one by one. There was no directional change in the overall HR, thus confirming that the results of this meta-analysis were robust and reliable. The test for heterogeneity for the primary outcome(PFS comparison between V3 and V1) indicated moderate to high heterogeneity (I²=42.9%). This heterogeneity was partially explained by the subgroup analyses. The funnel plot generated for this comparison was visually roughly symmetrical (**Figure 9**), and Egger’s test result was not statistically significant (p=0.116), suggesting a low probability of significant publication bias.

## 4 Discussion

This meta-analysis systematically evaluated the impact of different ALK variants on the efficacy of ALK TKI therapy. The results indicated that the EML4-ALK V3 variant was associated with shorter PFS in patients with ALK-positive NSCLC receiving TKI treatment. An in-depth subgroup analysis was conducted for this core comparison. First, we performed a subgroup analysis based on the type of ALK TKI used. Although this meta-analysis included studies with data on alectinib treatment, these studies reported efficacy outcomes using different variant classification methods, such as “long fusion variants vs. short fusion variants” or “V1 vs. non-V1”. Studies containing treatment data on ensartinib and lorlatinib were also included in this review. However, given that only one study was included for each, subgroup analysis was not performed. The results showed that in the crizotinib-treated subgroup, the difference in PFS between V3 and V1 reaches statistical significance (11, 13, 19–21, 24, 30, 35, 39). In the brigatinib-treated subgroup, the pooled effect size indicated that the V3 patients had more than twice the risk of disease progression compared with the V1 patients (HR = 2.09). But we must admit that this finding is based on only two studies (23, 24). Such a small number of studies will reduce statistical power and make the pooled estimate less stable and potentially over-influenced by individual study results. Future validation in larger studies is urgently needed to further clarify the true prognostic value of the V3 variant, particularly in the context of treatment with newer generation ALK TKIs. Next, analysis by treatment line revealed that in the first-line treatment subgroup, V3 showed a statistically significant PFS disadvantage with low heterogeneity, suggesting that the negative effect of V3 may be more pronounced in treatment-naive patients (11, 13, 24, 35, 39). In the all lines treatment subgroup, the effect size did not reach statistical significance (19–21, 23, 30, 34, 40, 41). This result may be related to clinical heterogeneity among studies, such as differences in the proportion of patients receiving later-line therapy and prior TKI exposure. Finally, the analysis regarding detection methods provides direction for future research. After excluding studies that used mixed detection methods, we found that in studies using only NGS, V3 was significantly associated with worse PFS, with no significant heterogeneity within the subgroup (11, 13, 20, 21, 23, 24, 34, 39–41). In contrast, no significant difference was observed in the non-NGS subgroup (19, 35). This finding may reflect the higher accuracy of NGS for variant subtyping, but it could also be due to the small number of studies in the non-NGS subgroup.

Furthermore, when comparing V3 with non-V3 variants, V3 showed a significant PFS disadvantage (18, 26, 33, 36, 37). When comparing V1 with non-V1 variants, V1 was associated with significantly longer PFS (14, 17, 26, 27). After excluding V3 from the non-V1 group, the comparison between V1 and other variants no longer showed a significant difference (19, 22). This finding suggests that among non-V1 variants, V3 may be the main driver of poor prognosis, while the clinical impact of other variants (such as V2) may be relatively limited. In addition to the comparisons based on specific variants (V1, V2, V3), we also attempted to explore the prognostic difference between long and short fusion variants from the perspective of fusion fragment length. The results showed that although long fusion variants tended to be associated with PFS benefit, this did not reach statistical significance (14, 16, 25, 32). Some previous studies have suggested that longer EML4 fragments may retain more microtubule binding domains, making the fusion protein more dependent on EML4 function and potentially more sensitive to ALK TKI treatment (10). The negative finding in this analysis may be related to the limited number of included studies.

Regarding secondary outcomes, although V3 showed a trend toward an increased risk of worse OS compared with V1, this did not reach statistical significance (20, 21, 30, 31, 34, 35). This may be related to the fact that OS data are often confounded by factors such as subsequent treatment crossover and loss to follow-up. Additionally, the follow-up duration in most included studies was insufficient to fully assess survival differences. In terms of ORR, no significant difference was observed between V1 and V3 (13, 19, 24, 29, 39, 41), suggesting that the negative effect of V3 may be primarily reflected in accelerating the development of acquired resistance rather than affecting initial tumor response. This finding is consistent with the biological characteristic that V3 is more prone to inducing resistance mutations such as G1202R (42, 43).

From the perspective of biological mechanisms, the potential reasons for the poorer efficacy of ALK TKIs associated with EML4-ALK V3 involve multiple aspects: Firstly, compared to V1, V3 exhibits higher protein stability and a greater tendency to form droplet-like subcellular structures, which may contribute to enhanced metastatic potentia^l^ (10, 42, 44, 45). Secondly, V3 is more prone to developing resistance mutations, such as G1202R, which can impair the effectiveness of ALK TKIs (42, 43). Finally, V3 shows a certain association with TP53, and this co-mutation of genes may lead to a poorer prognosis (42, 43).

From a clinical perspective, the findings of this study should be interpreted within the context of the evolving treatment landscape for ALK-positive NSCLC. Most of the data in this study were derived from patients treated with crizotinib, while prospective data regarding newer generation ALK TKIs such as alectinib and lorlatinib are extremely limited. In recent years, analysis from the CROWN trial has shown that lorlatinib still demonstrates significant PFS benefit in the V3 variant subgroup, with a magnitude of benefit comparable to that in the non-V3 subgroup, suggesting that newer generation ALK TKIs may partially overcome the negative prognostic effect of V3 (46). Therefore, the conclusions of this study mainly reflect the characteristics of crizotinib, and the prognostic value of V3 for drugs currently widely used in clinical practice, such as alectinib and lorlatinib, needs to be reassessed in prospective cohorts.

In summary, this meta-analysis has several limitations: (1) Most of the included studies were retrospective in nature, representing a lower level of evidence with potential risks of selection bias, information bias, and residual confounding. (2) Key prognostic factors, such as TP53 co-mutation status, baseline brain metastases, liver metastases, and number of prior treatment lines, were not uniformly adjusted for across studies. Most studies reported either unadjusted hazard ratios or those adjusted for only a limited set of variables, which may lead to bias in effect estimates. (3) The main comparison (V3 vs. V1) and the subgroup analyses all showed varying degrees of heterogeneity, indicating clinical heterogeneity among studies in terms of patient characteristics, treatment regimens, and outcome definitions, which limits the stability of the pooled results. (4) Among the 30 studies included in this analysis, the majority were based on patient populations treated with crizotinib, while data regarding second-generation and third-generation ALK TKIs were relatively limited, restricting the timeliness and clinical relevance of the conclusions. Future larger-scale, prospective studies with validation based on individual patient data are needed.

## 5 Conclusion

This meta-analysis confirms that EML4-ALK V3 is associated with shorter PFS in patients with ALK-positive NSCLC receiving TKI therapy. Subgroup analysis showed that the negative prognostic effect of V3 reached statistical significance in the crizotinib-treated subgroup, first-line treatment subgroup and in the subgroup using NGS for variant detection. A significant association was also observed in the brigatinib-treated population; however, this finding was based on only two studies, and the strength of evidence is limited, warranting cautious interpretation. However, the strength of this conclusion is limited due to the retrospective nature of the included studies, heterogeneity among studies, and inadequate control for confounders. Before recommending routine clinical stratification based on ALK variant type, prospective clinical studies with large sample sizes and standardized molecular subtyping are needed, particularly in populations treated with newer generation ALK TKIs, to clarify the true prognostic value of EML4-ALK V3 in the contemporary treatment landscape.

## Data Availability

The original contributions presented in the study are included in the article/supplementary material. Further inquiries can be directed to the corresponding author.
